# Association analysis of apoptosis-related gene caspase3, Integrin a subunit 1 and glutathione sulfur transferase M1 gene polymorphisms and susceptibility to gastric cardia carcinoma

**DOI:** 10.5937/jomb0-37763

**Published:** 2023-08-25

**Authors:** Rui Wang, Zetian Zhang, Duo Li, Na Wu, Zhao Peng

**Affiliations:** 1 The First Affiliated Hospital of Hebei North University, Department of Gastroenterology, Zhangjiakou City, Hebei Province, China

**Keywords:** gastric cardia cancer, apoptosis-related gene caspase3, Integrin a subunit 1, glutathione thitransferase M1, gene polymorphism, susceptibility, karcinom kardije želuca, GEN caspase3 povezan sa apoptozom, integrin a podjedinica 1, glutation titransferaza M1, polimorfizam gena, osjetljivost

## Abstract

**Background:**

To explore the association of polymorphisms of apoptosis-linked genes caspase3 (CASP3), integrin a subunit 1 (ITGA1), glutathione sulfur transferase M1 (GSTM1) with susceptibility to gastric cardia carcinoma (GCC).

**Methods:**

From February 2016 to March 2018, selection of 113 GCC patients was as the gastric cancer (GC), and selection of 75 patients without gastric disease was as the control. Detection of CASP3, ITGA1 and GSTM1 gene polymorphisms in patients' peripheral blood was to analyze their association with GC. Division of the GC was into the good prognosis and the unpleasing prognosis in the light of the survival of patients after surgery of 3 years, and the predictable value of gene polymorphisms of CASP3, ITGA1 and GSTM1 in GCC patients was analyzed.

**Results:**

CASP3 gene rs12108497 locus, ITGA1 gene rs1862610 locus and GSTM1 genotype of the GC and the control were in accord with Hardy-Weinberg equilibrium (P > 0.05); The detection rate of CASP3 gene rs12108497 locus TC/CC type, ITGA1's gene rs1862610 locus AC/AA type and GSTM1 blank type in the GC was elevated vs. the control (P < 0.05); Logistic regression analysis manifested smoking, anxiety, helicobacter pylori infection, family history of gastrointestinal tumor, combination with chronic gastric disease, CASP3 gene and GSTM1 gene polymorphism were risk factors for GC (P < 0.05); Stratification was in the light of individual smoking status, discovering that the detection rates of CASP3 gene rs12108497 locus TC/CC type, ITGA1 gene RS1862610 locus AC/AA type and GSTM1 blank type in the smoking were crucially augmented vs. the smoking (P < 0.05); The detection rates of CASP3 gene rs12108497 locus TC/CC type, ITGA1 gene rs1862610 locus AC/AA type and GSTM1 blank type in the death were augmented vs. the survival (P < 0.05); Combined detection of CASP3, ITGA1 and GSTM1 gene polymorphisms was provided with predictive value for GCC's prognosis (P < 0.05).

**Conclusions:**

CASP3 and GSTM1 genes are susceptibility genes for GCC, which might be associated with the occurrence of GCC in smoking patients, and the joint detection of multiple genes is provided with predictive value for patients' prognosis.

## Abstract (sr)

**Uvod:**

Cilj je bio da se istraži povezanost polimorfizama gena povezanih sa apoptozom kaspaze3 (CASP3), integrin a podjedinicom 1 (ITGA1), i glutation sumpor transferazom M1 (GSTM1) sa osetljivošću na karcinom kardije želuca (GCC).

**Metode:**

Od februara 2016. do marta 2018. selekcija 113 pacijenata sa GCC je bila kao karcinom želuca (GC), a kao kontrola bila je selekcija 75 pacijenata bez bolesti želuca. Otkrivanjem polimorfizma gena CASP3, ITGA1 i GSTM1 u perifernoj krvi pacijenata trebalo je da se analizira njihova povezanost sa GC. Podela GC je bila na dobru prognozu i neprijatnu prognozu u svetlu preživljavanja pacijenata posle operacije od 3 godine, a analizirana je i predvidljiva vrednost polimorfizma gena CASP3, ITGA1 i GSTM1 kod pacijenata sa GCC.

**Rezultati:**

CASP3 GEN rs12108497 lokus, ITGA1 GEN rs1862610 lokus i GSTM1 genotip GC i kontrola bili su u skladu sa Hardi-Weinberg-ovom ravnotežom (P > 0,05); Stopa detekcije CASP3 gena rs12108497 lokusa TC/CC tipa, ITGA1 gena rs1862610 lokusa AC/AA tipa i GSTM1 praznog tipa u GC je bila povišena u odnosu na kontrolu (P <0,05); Logistička regresiona analiza manifestovala je pušenje, anksioznost, infekciju Helicobacter pilori, porodičnu anamnezu gastrointestinalnog tumora, kombinaciju sa hroničnom bolešću želuca, dok su CASP3 GEN i polimorfizam gena GSTM1 bili faktori rizika za GC (P < 0,05); Stratifikacija je bila u svetlu individualnog pušačkog statusa, otkrivajući da su stope detekcije CASP3 gena rs12108497 lokusa TC/CC tipa, ITGA1 gena RS1862610 lokusa AC/AA tipa i GSTM1 praznog tipa kod pušenja bile ključno povećane u odnosu na pušenje (P < 0,05); Stope detekcije CASP3 gena rs12108497 lokusa TC/CC tipa, ITGA1 gena rs1862610 lokusa AC/AA tipa i GSTM1 praznog tipa u smrti su bile povećane u odnosu na preživljavanje (P < 0,05); Kombinovana detekcija polimorfizama gena CASP3, ITGA1 i GSTM1 dala je prediktivnu vrednost za prognozu GCC-a (P <0,05).

**Zaključak:**

CASP3 i GSTM1 geni su geni osetljivosti na GCC, što može biti povezano sa pojavom GCC kod pušača, a zajedničko otkrivanje više gena ima prediktivnu vrednost za prognozu pacijenata.

## Introduction

Gastric cardia carcinoma (GCC) is the malignant tumor that happens at the junction of esophagus and stomach, meanwhile, the patients are not presented distinct symptom in the early phase, and the symptoms covering vomiting, deglutition difficulty are manifested in the advanced stage. Presently, genetic susceptibility is also linked with the occurrence of GCC, except for external factors [1]. Relevant reports have illuminated genes like GSTM1 are nearly associated with the occurrence and advancement of multiple malignant tumors [2]. Glutathione-s-transferases M1 (GSTM1), a group of isoenzymes, is nearly linked with the metabolic process of exogenous compounds, which is available to catalyze the electrophilic binding of Glutathione (GSH) sulfhydryl group with carcinogenic activity generated via the metabolic activation of various enzymes, thereby forming hydrophilic substances excreted from the body, and strengthening foreign compounds’ detoxification [3]. Caspase3 is a proteolytic enzyme of mediating cell apoptosis, and its family members exert a critical role in the cell apoptosis [4].

Apoptosis-related gene Caspase3 (CASP3), belonging to critical member of the Caspase family, exerts a regulatory role in the initiation and execution of cell apoptosis. Integrins, a complete membrane receptor family of existing on the surface of eukaryotic cells, are available to mediate the adhesion of cells to extracellular matrix and enable cells to attach to form a whole. Integrins 1 (ITGA1), a member of the cell adhesion receptor family, is an extremely crucial receptor protein, which is the major way for cells to combine with extracellular matrix and respond, and exerts the greatly critical action in a sequence of crucial physiological and pathological processes like cell growth, malignant tumors and wound healing [5]. Nevertheless, no reports have clarified association of it with GC’s susceptibility so far. Consequently, this study was to explore the association of CASP3, ITGA1 and GSTM1 genes polymorphisms with susceptibility to GCC, which was linked with the occurrence and development of this disease.

## Materials and methods

### Clinical data

From February 2016 to March 2018, enrollment of 113 GCC patients was as the GC, and enrollment of 75 patients without gastric disease was as the control during the identical period. Inclusion criteria: 1. The GC met the diagnostic criteria of GCC in *Standardized Diagnosis and Treatment Guidelines for Gastric Cancer* (Trial) [6]; 2. Age ≥ 18 years; 3. Complete cli nical data. Exclusion criteria: 4. Patients with severe cardiac, hepatic and renal abnormalities; Patients with combination of other primary malignant tumor diseases; Patients with combination of esophageal or intestinal malignant lesions; Patients with recent adoption of antibiotics or non-steroidal anti-inflammatory drugs; 5. Non-GCC patients; 6. Patients with combination of autoimmune diseases.

### Clinical data collection

Inquiry of patients’ general information covering age, gender, BMI, marital status, education background, family per capita monthly income, etc. was via adopting the hospital information system. BMI: 18.5–23.9 kg/m^2^ was normal, < 18.5 kg/m^2^ or > 23.9 kg/m^2^ was abnormal. Anxiety was self-rating anxiety Scale (SAS) > 50 points.

### Detection of CASP3, ITGA1 and GSTM1 genes polymorphisms

Collection of 3 mL fasting venous blood was from the patients, and extraction of peripheral bloodgenomic DNA was via adopting the Blood Genomic DNA extraction kit (Beijing Tiangen Company).Genotyping was implemented exerting TaqMan method. Design and synthesis of primers, FAM andVIC labeled probes was performed (Applied Biosystems). Primer design was via exerting PrimerPremier 5 software. CASP3 gene polymorphism: CASP3 gene RS12108497 locus: Upstream and downstream primers were 5‘-AGCCGCTCACGCATCATAGT-3’ and 5‘-AGTCCGCATCCAGCCAGGTA-3’, separately, and the length of the target fragment was 317 bp. ITGA1 gene: 5‘-CTGGGATTGTGGA AGGAGGG-3’ and 5‘-TGCTGTAAACATTGGGTCG-3’, 462 bp. GSTM1 gene: 5‘-GaactCCCTGAAAA GCTAAAGC-3’ and 5‘-GTTGGGCT CA AA TATACGGTGG-3’. The missing 215 bp fragment was blank type.

### Prognosis grouping

Division of patients was into the death and the survival on the grounds of their survival after cure.

### Observation indexes

(1) Analysis of the clinical data and CASP3, ITGA1 and GSTM1 gene polymorphisms in peripheral blood of the GC and the control was to analyze GCC’s risk factors. (2) Division of the GC was into the good prognosis and the unpleasing prognosis in the light of the 3-year postoperative survival to analyze the predictive value of CASP3, ITGA1 and GSTM1 gene polymorphisms for GCC patients’ prognosis.

### Statistical processing

Processing of the data was via exerting SPSS22.0 software. Manifestation of the enumeration data was in %, and comparison of the difference between groups was via x^2^ test. Manifestation of the measurement data was in mean ± standard deviation (SD) after normal test, and comparison of the difference between groups was via t test. Analysis of GCC’s risk factors was via adopting logistic regression. Analysis of the prognostic value of CASP3, ITGA1 and GSTM1 gene polymorphisms in GCC patients was via adopting receiver operating characteristic (ROC) curve. *P* < 0.05 was accepted as indicative of distinct differences.

## Results

### Hardy-Weinberg balance analysis of CASP3 and ITGA1 genes in the GC and the control

The genotypes of CASP3 gene rs12108497 locus and ITGA1 gene rs1862610 locus in the GC and the control were on the grounds of Hardy-Weinberg balance (*P* > 0.05), as presented in Table 1.

**Table 1:** Hardy-Weinberg equilibrium analysis of CASP3 and ITGA1 genes in the GC and the control.

Genes		The gastric cancer<br>(n = 113)		The control<br>(n = 75)	
		Actual<br>frequency	Theoretical<br>frequency	Actual<br>frequency	Theoretical<br>frequency
CASP3 gene<br>Rs12108497<br>locus	TT	37	35.12	41	41.07
	TC	52	55.75	29	28.86
	CC	24	22.12	5	5.07
ITGA1 gene<br>Rs1862610 locus	CC	25	26.77	30	30.72
	AC	60	56.46	36	34.56
	AA	28	29.77	9	9.72

### Single-factor analysis of GCC’s occurrence

In the GC, the proportion of patients with aberrant BMI, smoking, anxiety, helicobacter pylori (HP)infection, family history of gastrointestinal tumor, combination with chronic gastropathy, CASP3 gene rs12108497 locus TC/CC type, ITGA1 gene rs1862610 locus AC/AA type, GSTM1 gene blank type was elevated vs. the control (*P *< 0.05), as manifested in Table 2.

**Table 2:** Univariate analysis of GCC (cases).

Factors		The GC<br>(n = 113)	The control<br>(n = 75)	x^2^	P
Gender	Male	71	45	0.153	0.696
	Female	42	30		
Age (Years)	60 or more	67	36	2.321	0.128
	Less than 60	46	39		
BMI	Abnormal	59	28	4.014	0.045
	Normal	54	47		
Marital status	Married	59	35	0.555	0.456
	Unmarried or divorced	54	40		
Education	Junior high school and below	39	25	0.028	0.867
	High school and above	74	50		
Per capita monthly household income<br>(Yuan)	3000 or more	62	36	0.852	0.356
	Less than 3000	51	39		
Place of residence	Rural area	26	13	0.883	0.347
	Town	87	62		
Alcohol drinking	51	28	1.126	0.289	
Smoking	51	15	12.499	< 0.000	
Anxiety	38	7	14.614	0.000	
HP infection	43	12	10.593	0.001	
Family history of gastrointestinal tumors	11	1	5.325	0.021	
Combination with chronic gastric disease	19	3	7.164	0.007	
CASP3 gene rs12108497 locus	TT type	37	41	8.925	0.003
	TC/CC type	76	34		
ITGA1 gene Rs1862610 locus	CC type	25	30	6.960	0.008
	AC/AA type	88	45		
GSTM1 gene	Wide-type (WT)	37	46	14.944	< 0.001
	Blank type	76	29		

### Multi-factor analysis of GCC’s occurrence

Logistic regression analysis elucidated smoking, anxiety, HP, family history of gastrointestinal tumor, combination with chronic gastric disease, of CASP3 and GSTM1 gene polymorphisms were risk factors for GC (*P* < 0.05), as manifested in Table 3.

**Table 3:** Multi-factor analysis of GCC. Assignment: BMI (abnormal = 1, normal = 0); Smoking (yes = 1, no = 0); Anxiety (yes = 1, no = 0); HP infection (yes = 1, no = 0); Family history of gastrointestinal tumor (yes = 1, no = 0); Combination with chronic gastric disease (yes = 1, no = 0); CASP3 rs12108497 locus (TC/CC type = 1, TT type = 0); Rs1862610 locus of ITGA1 gene (AC/AA type = 1, CC type = 0); GSTM1 gene (blank type = 1, WT = 0).

Indexes	β	SE	Wald x^2^	OR	95% CI	P
BMI	0.412	0.238	2.997	1.510	0.947 2.407	~ 0.084
Smoking	0.641	0.172	13.889	1.898	1.355 2.659	~ < 0.001
Anxiety	0.595	0.169	12.395	1.813	1.302 2.525	~ < 0.001
Hp infection	0.831	0.328	6.419	2.296	1.207 4.366	~ 0.012
Family history of gastrointestinal tumors	0.686	0.201	11.648	1.986	1.339 2.945	~ 0.001
Combination with chronic gastric disease	0.537	0.175	9.416	1.711	1.214 2.411	~ 0.002
CASP3 gene rs12108497 locus	0.685	0.229	8.948	1.984	1.266 3.108	~ 0.003
ITGA1 gene rs1862610 locus	0.414	0.193	4.601	1.513	1.036 2.208	~ 0.033
GSTM1 gene	0.479	0.295	2.636	1.614	0.906 2.878	~ 0.105

### Comparison of CASP3, ITGA1 and GSTM1 gene polymorphisms in the smoking and the nonsmoking

The detection rates of CASP3 gene rs12108497 locus TC/CC type, TGA1 gene rs1862610 locusAC/AA and GSTM1 blank type in the smoking were distinctly elevated vs. the non-smoking (*P* < 0.05) (Table 4).

**Table 4:** Comparison of CASP3, ITGA1 and GSTM1 gene polymorphisms of the smoking with the non-smoking.

Genes		The smoking<br>(n = 51)	The non-smoking<br>(n = 62)	x^2^	P
CASP3 gene<br>rs12108497 locus	TT	12	25	11.529	0.003
	TC	21	31		
	CC	18	6		
ITGA1 gene Rs1862610<br>locus	CC	15	10	14.909	0.001
	AC	17	43		
	AA	19	9		
GSTM1 gene	WT	5	32	22.211	< 0.001
	Blank type	46	30		

### Comparison of CASP3, ITGA1 and GSTM1 gene polymorphisms of the death and the survival

The 3-year survival rate of GC patients was 66.37% (75/113). The detection rates of CASP3 gene rs12108497 locus TC/CC type, ITGA1 gene rs1862610 AC/AA type and GSTM1 blank type were augmented vs. the survival (*P* <0.05), as presented in Table 5.

**Table 5:** Comparison of CASP3, ITGA1 and GSTM1 genes polymorphisms in the death and the survival.

Genes		The death<br>(n = 38)	The survival<br>(n = 75)	x^2^	P
CASP3 gene<br>Rs12108497 locus	TT	5	32	23.726	< 0.001
	TC	13	39		
	CC	20	4		
ITGA1 gene<br>Rs1862610 locus	CC	4	21	9.771	0.008
	AC	17	43		
	AA	17	11		
GSTM1 gene	WT	4	33	9.973	0.002
	Blank type	34	42		

### Analysis of the predicative value of joint examination of CASP3, ITGA1 and GSTM1 gene polymorphisms for GCC’s prognosis

**Figure 1:**
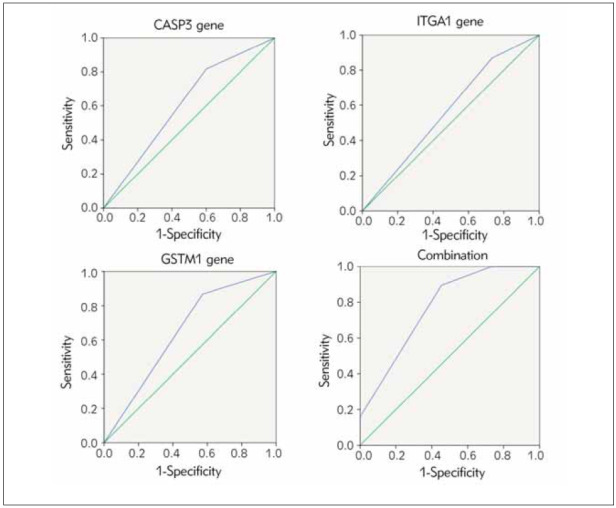
Coordinate curves for disease activity parameters and ROC curve showing Area under the Curve (AUC) of u/NGAL, proteinuria 24h, SLEDAI/r Up/k, anti ds DNA Ab, ANA.

Predictive value of combined detection of CASP3, ITGA1 and GSTM1 gene polymorphisms forGCC’s prognosis (*P* < 0.05), as manifested in Table 6 and Figure 1.

**Table 6:** Predictive value of combined detection of CASP3, ITGA1 and GSTM1 gene polymorphisms for GCC’s prognosis. Vs. the combination, ^*^ P < 0.05.

Genes	AUC	SE	95% CI
CASP3 gene rs12108497 locus	0.648^*^	0.053	0.545~0.751
ITGA1 gene rs1862610 locus	0.587^*^	0.055	0.480~0.695
GSTM1 gene	0.667^*^	0.051	0.567~0.768
Joint examination	0.771	0.044	0.685~0.856

## Discussion

Recently, the global incidence of GC has declined promptly, but the incidence of GCC is increasing with each passing year all over the world [7]. Presently, domestic epidemiological studies have illuminated the incidence of GCC has also elevated in China [8]. Researches have illuminated genetic susceptibility is associated with GCC’s occurrence and development [9]. CASP3, a critical apoptosis-linked gene, is located in the 4q35.1 region of chromosome, and is available to be activated via CASP9 or itself cleaved into two small molecule end-peptides during apoptosis, which is available to act on mitochondriamediated internal apoptosis pathway. Relevant researches adopted Alibaba2 software to predict that this polymorphic variation might eliminate the binding site of one GATA binding protein on CASP3 gene, thereby influencing gene transcription and selective splicing. Consequently, rs12108497 locus polymorphism is nearly associated with CASP3 gene, and rs12108497 locus-mediated ASP3 gene is linked with its location in CASP3 promoter region as well [10].

ITGA1, a receptor protein in the form of heterodimer, mediates the interaction between cells and between cells and cell matrix [11] [12]. ITGA1 is available to combine with tyrosine kinase FYN via the concave protein, thereby aggregating growth factor receptor-binding protein 2 to activate Ras/ERK pathway and modulating cell proliferation. GST, a group of multifunctional proteins, is available to catalyze the binding of reduced GSH to electron-friendly harmful compounds and expel various endogenous and exogenous potentially toxic compounds from the body in a non-enzymatic form. GSTM1 is a critical enzyme of catalyzing phase reaction in GST biotransformation reaction, and the maintenance of its activity is of dramatical value for the elimination of multiple carcinogens. Several relevant researches have illuminated GSTM1 gene polymorphism is nearlyassociated with the occurrence of cancers like GC and uterine cancer [12] [13]. Based on this, this study analyzed the association of GSTM1 gene polymorphism with GCC, and the results clarified the detection rates of CASP3 gene rs12108497 locus TC/CC type, ITGA1 gene rs1862610AC/AA type and GSTM1 blank type in the GC were augmented vs. the control, manifesting that the aberrant rate of CASP3, ITGA1 and GSTM1 gene polymorphisms in GCC patients were augmented, and suggesting that CASP3, ITGA1 and GSTM1 gene polymorphisms in GC patients might be associated with GC’s occurrence and advancement. Logistic regression analysis has elaborated smoking, anxiety, HP infection, family history of gastrointestinal tumor, combination with chronic gastric disease, CASP3 and GSTM1 gene polymorphisms are risk factors for GC, and the reasons are as follow: GSTM1 is available to influence the detoxification ability of individuals to environmental carcinogens, elevated ITGA1 is available to augment gastrointestinal mucosa inflammation, CASP3 is available to mediate cell apoptosis, and multi-gene interaction is available to further boost GC’s occurrence [14].

Whether smoking and genetic susceptibility are provided with the combined effect in GC’s advancement has also become a research hotspot among GC’s risk factors. Numerous researches have clarified smoking is nearly associated with GC’s occurrence [15] [16]. Recently, studies have illuminated smoke contains free radicals, which is available to destroy genetic genes, damage cell membranes and reduce immune function, thus leading to cancer’s occurrence [17] [18]. Consequently, the author maintains smoking might influence GCC’s occurrence via impacting gene. In this research, stratification was conducted in line with the individual smoking status, which manifested the detection rates of CASP3 gene rs12108497 locus TC/CC type, ITGA1 gene rs1862610 locus AC/AA type and GSTM1 blank type in the smoking were distinctly elevated vs. the nonsmoking, illuminating that CASP3, ITGA1 and GSTM1 gene polymorphisms in GCC patients might be associated with smoking, and the reasons are uncertain presently. Consequently, further analysis is required in the later stage.

Currently, the research has elucidated gene polymorphism exerts a certain role in GC’s growth and metastasis. CASP3 is an effector of the Caspase apoptosis cascade pathway and exerts a critical action in both external and internal apoptosis [19] [20]. GSTM1, a member of the Mu family, is available to catalyze the initial step of GSH binding, and is the critical enzymes in biological transformation of the body, exerting a crucial role in the metabolic environment carcinogens. GSTM1 gene polymorphism is available to impact carcinogens’ metabolism, and then influence cancer cells’ growth [21] [22], clarifying that it might be associated with patients’ prognosis. The results of this study illuminated the detection rates of CASP3 gene rs12108497 locus TC/CC type, ITGA1 gene rs1862610 locus AC/AA type and GSTM1 blank type in the death were elevated vs. the survival, elaborating that CASP3, ITGA1 and GSTM1 gene polymorphisms are linked with GCC’s prognosis. This research has discovered combined detection of CASP3, ITGA1 and GSTM1 gene polymorphisms is provided with predictive value for GCC’s prognosis, clarifying that detection of CASP3, ITGA1 and GSTM1 gene polymorphisms is available to be adopted to early assess GCC patients’ prognosis. This might be linked with the involvement of these three genes in cell advancement [23] [24].

In brief, CASP3 and GSTM1 genes were susceptibility genes for GCC’s occurrence, which might be associated with the occurrence of GCC in smoker patients, and the joint detection of multiple C genes was provided with predictive value for patients’ prognosis.

## Dodatak

### Acknowledgments

Not applicable.

### Funding

Not applicable.

### Conflict of interest statement

All the authors declare that they have no conflict of interest in this work.
